# Transcatheter embolization of the esophagomediastinal fistula with *N*-butyl cyanoacrylate glue: A case report

**DOI:** 10.1016/j.ijscr.2019.10.054

**Published:** 2019-10-29

**Authors:** Suk Hyun Bae

**Affiliations:** Department of Radiology, Inje University College of Medicine, Ilsan Paik Hospital, 170 Juhwa-ro, Ilsanseo-gu, Goyang, 10380, Republic of Korea

**Keywords:** Esophagomediastinal fistula, Esophageal perforation, *N*-Butyl cyanoacrylate, Embolization, Case report

## Abstract

**Introduction:**

Acute mediastinitis with esophageal perforation is a very fatal condition and must be treated promptly. Esophagomediastinal fistula is a rare complication of acute mediastinitis with esophageal perforation. There are many treatment options such as surgery or endoscopic treatment, but it is most important to start treatment immediately.

**Presentation of case:**

A 69-year-old female presented with chest pain and fever. Contrast enhanced chest computed tomography was compatible with acute mediastinitis and esophageal perforation. Esophagography revealed esophagomediastinal fistula in the upper esophagus. Endoscopic clipping with fibrin was failed and endoscopic vacuum therapy (EVT) was not effective for esophagomediastinal fistula. We performed the successful transcatheter embolization of the esophagomediastinal fistula with *N*-butyl cyanoacrylate (NBCA) glue.

**Discussion:**

There are many considerations in the treatment of acute mediastinitis with esophageal perforation, but surgery is the mainstay of treatment. Recently non operative management is appropriate in certain well-defined situations. Like our case patients, non-operative management may be considered if the diagnosis is delayed and the surgical treatment period is missed. Currently, endoscopic treatment such as covered stenting, clipping and application of fibrin glue are useful and a less invasive rather than surgical treatment. However, if endoscopic or surgical procedure are not possible, we considered transcatheter NBCA glue embolization.

**Conclusion:**

Transcatheter embolization with NBCA glue was proven to its effectiveness as an alternative therapeutic option in the treatment of esophagomediastinal fistula which endoscopic or surgical treatment are impossible or fails.

## Highlights

• Acute mediastinitis with esophageal perforation is a fatal disease.
• Esophagomediastinal fistula due to esophageal perforation is difficult to curable treatment.
• Successful embolization of the esophagomediastinal fistula with *N*-butyl cyanoacrylate.

## 1 Introduction

Perforation of esophagus is a very fatal disease entity with high mortality and morbidity [1]. Acute mediastinitis associated with esophageal perforation can result in life-threatening sepsis [2]. Therefore, treatment should be promptly initiated and operative management is the mainstay of treatment [1,2]. However, non-operative management is applicable to patients associated with well-contained perforations or delayed diagnosis [3].

Currently, endoscopic treatment such as covered stenting, clipping and application of fibrin glue are useful and a less invasive rather than surgical treatment. This is especially useful when esophagomediastinal fistula is caused by esophageal perforation [4].

Like our case, there is a need to find alternative treatment after endoscopic treatment failure for esophagomediastinal fistula. We report here the successful transcatheter embolization of the esophagomediastinal fistula of a 69 year old female with *N*-butyl cyanoacrylate (NBCA) glue.

Our institutional review board (IRB) does not require approval for case reports involving fewer than three patients.

This work has been reported in line with the SCARE criteria [5].

## 2 Case report

**Fig. 1:**
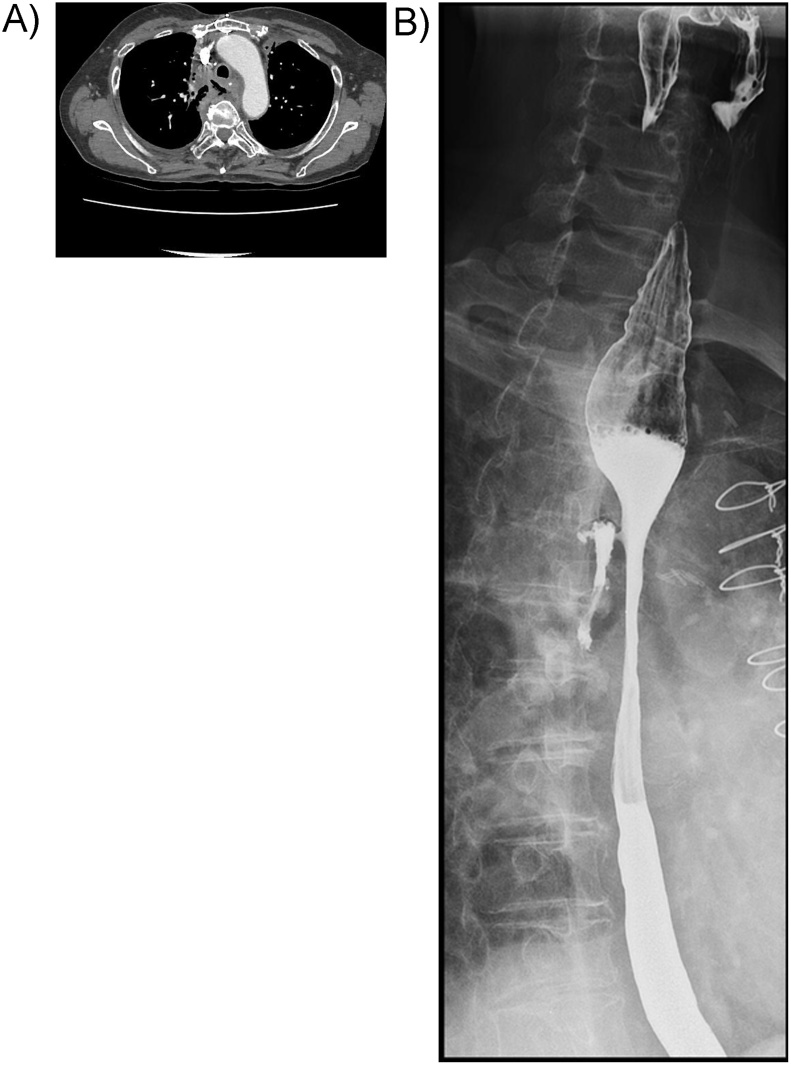
A: Enhanced chest computed tomography showing soft tissue infiltration with air-fluid collection around upper esophagus. B: Gastrografin esophagography showing esophagomediastinal fistula in the upper esophagus.

A 69 year old female visited emergency room for fever and chest pain. Contrast enhanced chest computed tomography (CT) revealed diffuse soft tissue infiltration with air-fluid collection around upper esophagus, which was compatible with acute mediastinitis and esophageal perforation (Fig. 1A). However, the diagnosis was missed and delayed at the time.

She received radiotherapy and chemotherapy for non-small cell lung cancer 17 years ago and was completely cured. And she underwent coronary artery bypass grafting (CABG) for acute myocardial infarction 7 years ago. Because of these underlying diseases and delayed diagnosis, the thoracic surgeon decided to perform conservative treatment rather than immediate surgery. Intravenous antibiotics and proton pump inhibitor were administered immediately.

Gastrografin esophagography, performed after 4 weeks, revealed esophagomediastinal fistula in the upper esophagus (Fig. 1B). Endoscopic vacuum-assisted closure therapy for esophagomediastinal fistula treatment was performed, but was not effective. Endoscopic clipping with fibrin was tried, but was not effective. So she was referred to our intervention department to treatment for esophagomediastinal fistula.

**Fig. 2:**
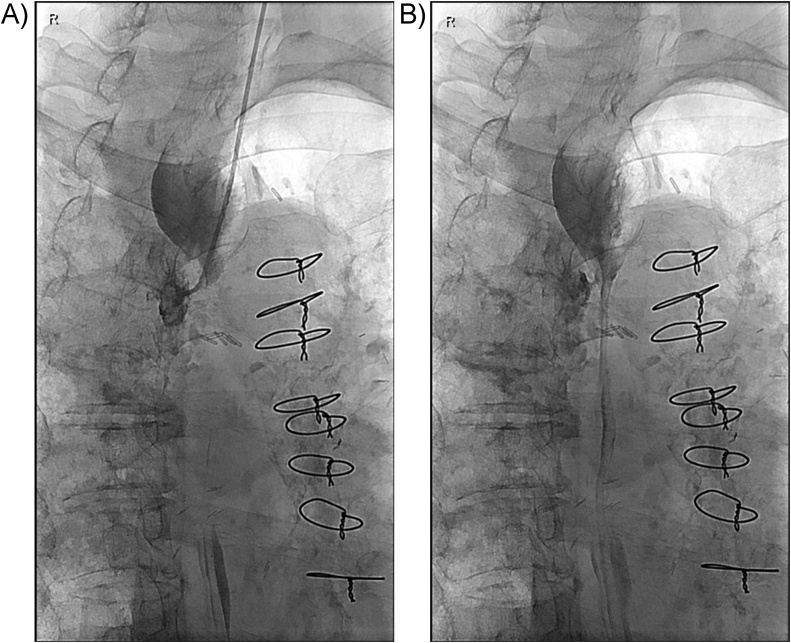
A: An orifice of the fistula was selected using the diagnostic angiographic catheter. B: Embolization of the fistula was performed by injection of the NBCA via the angiographic catheter.

We considered a fistula embolization with NBCA glue via transcatheter. A 4F angled taper Glidecath (Terumo Corporation, Shibuya-ku, Tokyo, Japan) was inserted into a nostril and passed into the upper esophagus. Esophagography was performed by injection of contrast material via diagnostic angiographic catheter. Esophagography revealed esophagomediastinal fistula in the upper esophagus. The diameter of the esophagomediastinal fistula was measured to be about 3 mm. Using the diagnostic angiographic catheter and 0.035 inch Glidewire (Terumo), an orifice of the fistula was selected. NBCA and iodized oil were mixed at a 1:3 ratio and 1.5 mL of the liquid glue was drawn up in a 3 mL syringe. Embolization of the fistula was performed by injection of the NBCA glue into the mediastinal soft tissue up to the orifice of the fistula via the diagnostic angiographic catheter (Fig. 2A & B).

**Fig. 3:**
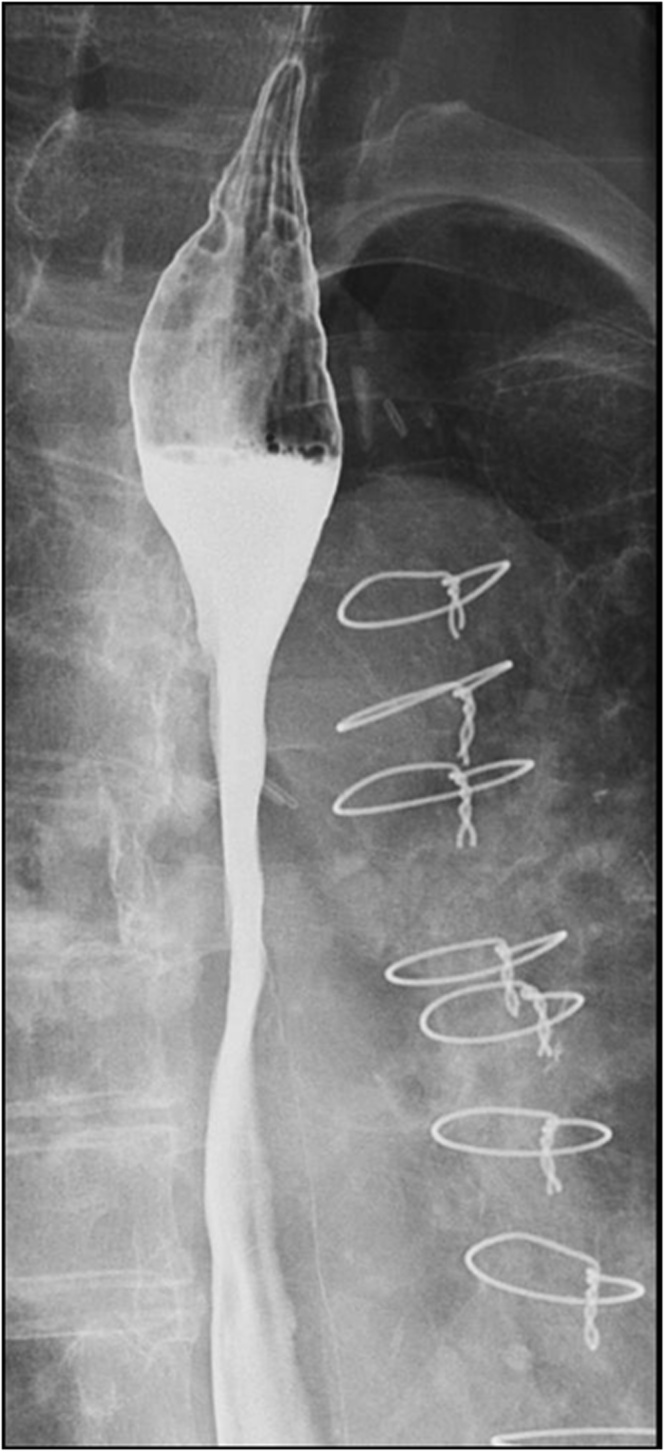
Follow-up esophagography after 4 weeks showing resolution of the esophagomediastinal fistula.

Esophagography after 4 weeks revealed resolution of the esophagomediastinal fistula (Fig. 3). At 2 months later after embolization, the patient’s condition has improved and she tolerates solid food. The patient was discharged without complications at 3 months after embolization. A follow-up esophagography after 6 months did not revealed esophageal fistula recurrence.

## 3 Discussion

Acute mediastinitis is a fatal infection which occurs related to connective tissue of the mediastinum. In recent studies, acute mediastinitis mortality has been estimated to range from 15.4% to 50% [6]. Acute mediastinitis often occurs with or is caused by esophageal perforation [2]. Esophageal perforation with acute mediastinitis can lead to sepsis and organ failure with very high mortality rate if diagnosis is missed or delayed [3]. In our case, acute mediastinitis developed by esophageal perforation required rapid treatment.

In determining the method of treatment for esophageal perforation, there are many things to consider including location, cause, general condition of the patient and the severity of the perforation [3]. But, first of all, treatment should be started as early as possible and that should include total parenteral nutritional support and intravenous broad spectrum antimicrobial therapy [3]. Surgery is the mainstay of treatment, but recently non operative management is appropriate in certain well-defined situations [1]. Like our case patients, non-operative management may be considered if the diagnosis is delayed and the surgical treatment period is missed.

There are several non-operative methods, including conservative management, esophageal stenting, endoclip application, endoscopic fibrin applications and endoscopic vacuum therapy [1,7]. Endoscopic vacuum therapy (EVT) is more effective for esophageal leakage because it makes rapid removal of necrotic debris or pus, and prevents further spread of contamination [8]. Also, Borejsza-Wysocki et al. reported that endoscopic vacuum-assisted closure system treatment may be an effective procedure and a routine choice for esophageal perforation [9]. In addition to EVT, endoscopic techniques such as stenting, clipping and fibrin injection have developed and useful [4,8]. Makino et al. reported that the endoscopic clipping with fibrin glue for esophagomediastinal fistula treatment is effective and not invasive [4]. However, our case patient attempted the EVT and endoscopic clipping with fibrin application, but the esophagomediastinal fistula persisted. We performed transcatheter glue embolization of the fistulous tract with NBCA.

NBCA is a cyanoacrylate derivatives that is commonly used as endovascular embolic agent for vascular malformation, hemorrhaging and venous disease. NBCA works instantly, completely occludes vessels, and is permanent [10]. In addition to endovascular embolic agents, there are case reports that NBCA is used as an embolic agent in bronchopleural, biliary and enteric fistulas [11,12]. However, NBCA glue embolization may be ineffective and may require a repeat procedure in the fistula of the nonvascular system [12].

We report the successful embolization of esophagomediastinal fistula by transcatheter NBCA glue embolization.

In conclusion, our case provide evidence that NBCA embolization is relatively noninvasive, safely, and cost-effective. In our case, we confirmed its effectiveness as an alternative therapeutic option in the treatment of esophagomediastinal fistula which endoscopic or surgical treatment are impossible or fails.

## Sources of funding

No funding has been declared by the authors.

## Ethical approval

The ethical approval for this case report is been exempt.

The submitted case report was not a study, therefore no ethical approval or informed written consent was needed.

## Consent

Written informed consent was obtained from the patient for publication of this case report and accompanying images. A copy of the written consent is available for review by the Editor-in-Chief of this journal on request.

## Author’s contribution

Dr. S.H. Bae — Study concept, Data collection, Author of case report and discussion, Review of literature.

## Registration of research studies

This is a case report. Hence, it is not registered in the clinical trial registry.

## Guarantor

Dr. S.H. Bae.

## Provenance and peer review

Not commissioned, externally peer-reviewed. No financial and personal relationships with other people or organisations that could inappropriately influence their work.

## Declaration of Competing Interest

No conflicts of interest declared by the authors.
